# See New Advertisement of Kidder & Laird

**Published:** 1881-11-20

**Authors:** 


See new advertisement of Kidder & Laird, commencing with this number of our Journal. The formula of Hydroleine is admirable.

